# The Role of Csmd1 during Mammary Gland Development

**DOI:** 10.3390/genes12020162

**Published:** 2021-01-26

**Authors:** Samuel J. Burgess, Hannah Gibbs, Carmel Toomes, Patricia L. Coletta, Sandra M. Bell

**Affiliations:** Leeds Institute of Medical Research, University of Leeds, St. James’s University Hospital, Leeds LS9 7TF, UK; umsbu@leeds.ac.uk (S.J.B.); H.Gibbs@leeds.ac.uk (H.G.); c.toomes@leeds.ac.uk (C.T.); p.l.coletta@leeds.ac.uk (P.L.C.)

**Keywords:** Csmd1, mammary gland development, epithelial mesenchymal transition, terminal end bud, extracellular matrix, Fak, Stat1, Akt

## Abstract

The Cub Sushi Multiple Domains-1 (CSMD1) protein is a tumour suppressor which has been shown to play a role in regulating human mammary duct development in vitro. *CSMD1* knockdown in vitro demonstrated increased cell proliferation, invasion and motility. However, the role of Csmd1 in vivo is poorly characterised when it comes to ductal development and is therefore an area which warrants further exploration. In this study a *Csmd1* knockout (KO) mouse model was used to identify the role of Csmd1 in regulating mammary gland development during puberty. Changes in duct development and protein expression patterns were analysed by immunohistochemistry. This study identified increased ductal development during the early stages of puberty in the KO mice, characterised by increased ductal area and terminal end bud number at 6 weeks. Furthermore, increased expression of various proteins (Stat1, Fak, Akt, Slug/Snail and Progesterone receptor) was shown at 4 weeks in the KO mice, followed by lower expression levels from 6 weeks in the KO mice compared to the wild type mice. This study identifies a novel role for Csmd1 in mammary gland development, with Csmd1 KO causing significantly more rapid mammary gland development, suggesting an earlier adult mammary gland formation.

## 1. Introduction

The CUB Sushi Multiple Domains-1 (*CSMD1*) gene is located in the chromosome region 8p23 spanning a 2MB region of DNA and this encodes multiple transcripts for the *CSMD1* gene in humans. The largest and most common form of the transcripts is 14,552 base pairs long and consists of 71 exons [1]. In total there are 17 splice variants of the *CSMD1* gene [2]. The largest of these variants encodes a 3565 amino acid protein which has a molecular mass of 383kDa in humans. The Csmd1 mouse protein demonstrates 93% homology to the human orthologue [1]. The CSMD1 protein primarily consists of 14 CUB domains and 14 sushi domains; these domains alternate so that each CUB domain is separated by a sushi domain, with a section of 14 sushi domains leading to the C-terminus [1]. The CUB (Complement C1r/C1s, Uegf, BMP1) domain is 110 residues in size and found in a range of extracellular and membrane bound proteins, with these proteins commonly having roles in developmental patterning and complement regulation [3,4,5]. The sushi domain, also known as the complement control protein domain, is 66 residues in size and is commonly associated with proteins involved in complement regulation or cell adhesion proteins [6,7,8]. The Csmd1 protein is located in the cell membrane, leading it to be determined as a transmembrane protein with a large N-terminal extracellular region and a short internal 66 amino acid cytoplasmic tail at the C-terminus which contains a putative tyrosine phosphorylation site [1]. CSMD1 has been shown to be expressed in both neuronal and epithelial tissues, with high levels of CSMD1 identified in the breast, testis and kidneys in humans and mice.

*CSMD1* has been characterised, using a range of in vivo and in vitro approaches, and is implicated in various processes, such as testicular development, complement regulation, schizophrenia and in cancer progression [9,10,11,12,13]. In relation to cancer, *CSMD1* has been identified as a tumour suppressor with previous studies identifying the chromosome location 8p23 as an area deleted in various cancers, such as breast, head and neck and colorectal [1,12,13,14,15,16,17,18,19]. Furthermore *CSMD1* deletion has also been linked to poor prognosis in patients with late stage breast cancer [12,17,20,21]. Recently, the role of Csmd1 in developmental processes was examined, with *Csmd1* knockout (KO) in mice leading to reduced fertility through altered regulation of spermatozoa production [11]. There have also been studies examining the role of Csmd1 and duct development. These studies show that pregnant mice have increased ductal growth when *Csmd1* is knocked out and in-vitro ductal studies identify more rapid cell growth and duct formation in *CSMD1* KO cells [11,22]. These studies, along with further characterisation studies of CSMD1, have identified that the deletion of CSMD1 causes increased cell invasion, motility and proliferation [14,15,22,23]. These studies therefore suggest a potential role for CSMD1 in the regulation of epithelial mesenchymal transition (EMT). EMT is the process by which functional epithelial cells are able to revert back to a stem cell state, which allows the cells to become more proliferative and migratory [24,25,26]. Furthering the connection between Csmd1 and EMT showed a connection between Csmd1 and Nf-κß, a well-known EMT factor, where *Csmd1* inhibition leads to increased Nf-κß activity and increased EMT phenotype in gastric cancer [14,27]. Along with changes in Nf-κß signalling, alterations in Smad signalling have also been identified to be associated with changes in Csmd1 expression [13,15,28]. Csmd1 has also been implicated in the regulation of complement, where it primarily regulates the formation of the C3 convertase [7,10,11,29]. Recently *Csmd1* KO has been shown to promote C3 deposition on the synapses promoting synaptic pruning in mice [29].

**Figure 1:**
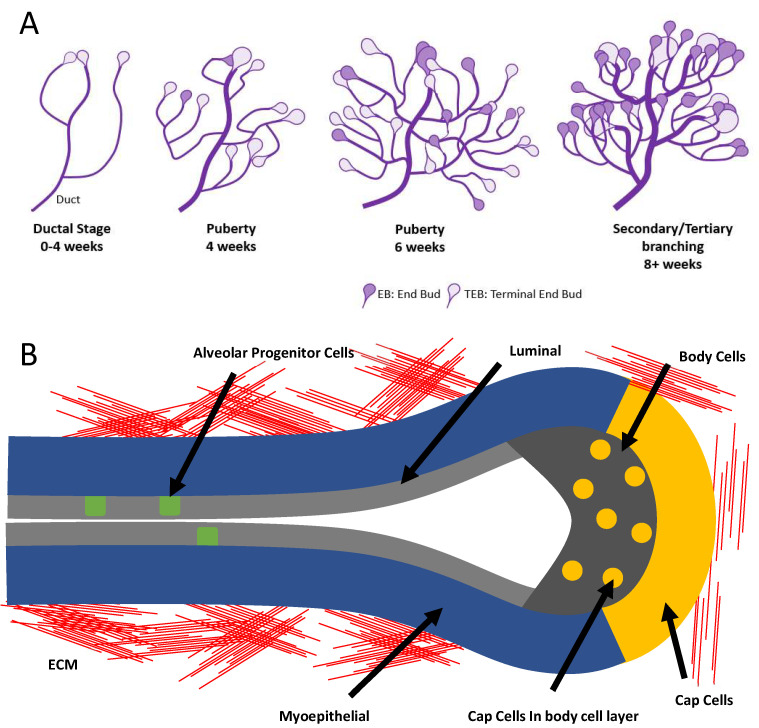
(**A**) Schematic of mammary gland development over the course of puberty (**B**) Terminal End Bud (TEB) structure, identifying the structure and types of cells present.

The mammary gland is responsible for producing milk, which is then required for the raising of new-borns as it provides them with all the nutrients required for development. Therefore the understanding of how the mammary gland develops is very important, as any abnormalities in mammary gland development will lead to malnourished and poorly developed new-borns. In mice the mammary gland undergoes most of its development during puberty between 4–8 weeks of age, with the gland being fully formed by 12 weeks of age (Figure 1A). During mammary gland development, the formation of the ductal tree is driven by structures called terminal end buds (TEBs) [30,31]. TEBs are highly proliferative structures at the ends of the ducts which promote ductal growth into the mammary fat pad leading to the formation of the ductal tree (Figure 1B). Csmd1 has been shown to be involved in the regulation of the ductal development in an in-vitro model [22]. This showed that *CSMD1* KO in MCF-10A cells is able to determine dysregulated ductal development by promoting increased cell proliferation, invasion and motility [22]. These characteristics were also shown in other in-vitro studies in breast cancer cell lines in which high levels of CSMD1 expression demonstrate slower motility and also decreased invasive properties compared to cell lines with none or inhibited CSMD1 expression [15,22].

Although some studies have briefly examined the role of Csmd1 in ductal development and also during pregnancy, currently no studies have fully explored the role of Csmd1 during mammary gland development. Consequently, this study used a *Csmd1* KO mouse model to examine the development of the mammary gland during puberty into adulthood, with the additional aim of identifying potential mechanisms of action for Csmd1. In this study specific protein expression patterns were analysed to determine changes *Csmd1* KO has on their expression. Proteins were selected primarily due to their connection to EMT regulation, a mechanism which Csmd1 has previously been suggested to be involved in regulating [14,15,22,23]. A range of proteins were analysed in order to narrow down the specific EMT pathway Csmd1 regulates.

## 2. Materials and Methods

### 2.1. Mouse Model

**Figure A1:**
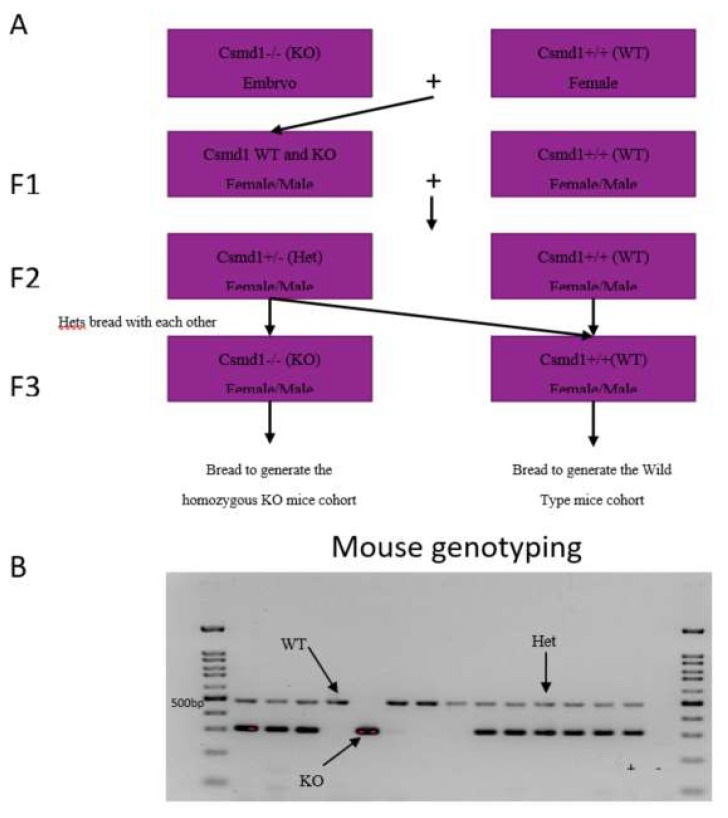
Information regarding the breeding structure used to generate the mouse cohort used in this study (**A**), along with a PCR gel electrophoresis image showing how the mice genotypes were identified (**B**). To generate the mouse the cohort initially a Csmd1 KO mouse embryos were implanted into a pregnant WT mouse, this generated the F1 generation consisting of both WT and KO mice. These mice were then breed with further WT mice (C57BL/6). This generated an F2 generation consisting of either heterozygotes (Hets) or WT mice. The Hets were then breed with each other to generate both KO and WT mice. The KO mice were then further breed with each other to obtain the full KO cohort, with mice being harvested at 4, 6, 8 and 12 weeks. The WT mice breed throughout this study were also harvested at the same time points.

The B6;129S5-Csmd1tm1Lex/Mmucd mouse strain (*Csmd1* KO) mouse was created by Lexicon Pharmaceuticals through deletion of a 1 kb genomic sequence composed of part of exon 1 and intron 1 caused by insertion of a LacZ/Neo cassette [32]. This *Csmd1* KO model generates a whole mouse knockout for Csmd1, including the spliced forms of Csmd1. *Csmd1* KO cryopreserved mouse embryos were first implanted into healthy wild type female (WT) mice (C57BL/6). This then created a F1 generation of heterozygotes, which were used to breed the F2 generation which has all 3 mouse types, with the F2 WT and KO mice being used to create the F3 generation of mice (Figure A1). New-born mice were then genotyped at 3 weeks of age by ear notch to identify which mice were WT, *Csmd1* KO or heterozygous mice. The F3 generation of female mice were then harvested at the required time points 4, 6, 8 and 12 weeks of age. At 4 weeks 14 WT and 14 KO mice; 6 weeks 9 WT and 11 KO mice; 8 weeks 7 WT and 8 KO mice; 12 weeks 7 WT and 9 KO mice were collected. The sample sizes were determined using the power analysis statistical test. All procedures were approved by the UK Home Office and carried out according to the Animals (Scientific Procedures) Act 1986.

### 2.2. Whole Mount Staining

The abdominal mammary glands were exercised from the female mice, with one gland being used for whole mount staining and the other embedded in paraffin to be used for staining. The gland used for the whole mount was fixed in 4% PFA overnight at room temperature (RT) (20 °C). The mammary gland was then rehydrated using decreasing concentrations of ethanol (100%, 70%, 50%) and dH_2_O. Following the rehydration the gland was submerged in carmine alum solution overnight at RT to stain the ducts in the mammary gland. After staining, the ducts were then dehydrated in increasing concentrations of ethanol (50%, 70%, 100%) and washed in xylene solution to remove any excess staining from the mammary gland, leaving only the ducts with visible staining. The glands were then mounted using a glycerol based mounting agent and were viewed and imaged at x4 objective using bright field capture on a Nikon Eclipse Ti-E microscope, this was to observe and count the TEBs. Images of the whole stained mammary gland were taken using a Sony digital SLR camera to obtain the area of the ductal tree.

### 2.3. Haematoxylin and Eosin (H&E) Staining

The mammary gland sections to be used for staining were initially fixed in 4% PFA overnight before being fixed in paraffin and then sectioned using a microtome at 5 µm. These sections were then used for the H&E, Picro-Sirus Red and IHC stains. For the H&E stain the sections were dewaxed using xylene before being rehydrated using a decreasing ethanol concentration before being submerged in Haematoxylin solution, followed by Eosin solution. After the staining with the two solutions, the sections were dehydrated using increasing ethanol concentrations and xylene. The sections were then mounted using DPX (Merek).

### 2.4. Picro-Sirus Red Staining

The sections were dewaxed and rehydrated as previously stated. The slides were then submerged in Wiegerts haematoxylin (Sigma) for 20 min to stain the nuclei. The slides were then stained using Picro-Sirus Red solution for 1 h, after which the slides were washed in tap water and left to dry for 30 min. Finally, the slides were dehydrated using 100% ethanol and then xylene, and then mounted using DPX.

### 2.5. Immunohistochemistry (IHC)

**Figure A2:**
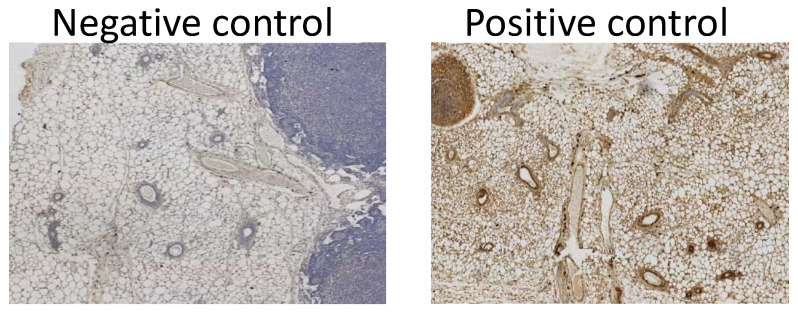
Image showing examples of the negative and positive controls used in this study. The negative control was an antibody free control to determine any background staining caused by the kit used. The positive control was performed on tissue known to express the protein in question. These tests were performed on the same tissue types for each run performed, with this tissue being full adult, >16 weeks of age, wild type mammary glands.

IHC was performed to analyse the expression of Mcm2, Stat1, Fak, Slug/Snail and Akt (Abcam) and progesterone receptor (Thermo Fisher). The paraffin embedded sections of mammary gland were stained using the primary antibodies following a previously described protocol using the conditions described in Table 1 [17]. Following the primary antibody incubation, staining was detected with Novolink polymer (Leica Biosystems) according to the manufacturer’s protocol. The sections were developed using diaminobenzidine (DAB) (Leica Biosystems) to visualise the areas of positive expression. Antibody free negative controls were used along with the use of tissues known to express the specific proteins as positive controls (Figure A2).

### 2.6. Quantification and Statistical Analysis

The quantification analysis for the Roundness and Picro-Sirus Red stains were performed using image J software. The Picro-Sirus Red stain used a threshold analysis to obtain the amount of collagen present around each duct, with each image being scaled to ensure all of the collagen had been selected for analysis. Roundness equation: 4 × area/(π x major_axis^2) (Image J manual). The analysis for the IHC markers used QuPath Software [33]. For this a script was created which was able to differentiate between cells with no/low levels of expression of the protein in question and those with high levels of expression, this would then provide the number of positive cells compared to the number of negative cells. This was performed for both nuclear and cytoplasmic expressing proteins. All *p*-values were obtained using the student’s t-test. Statistical tests *p* < 0.05 were considered significant.

## 3. Results

### 3.1. Csmd1 KO Leads to Increased Mammary Gland Development during Early Stages of Puberty

**Figure 2:**
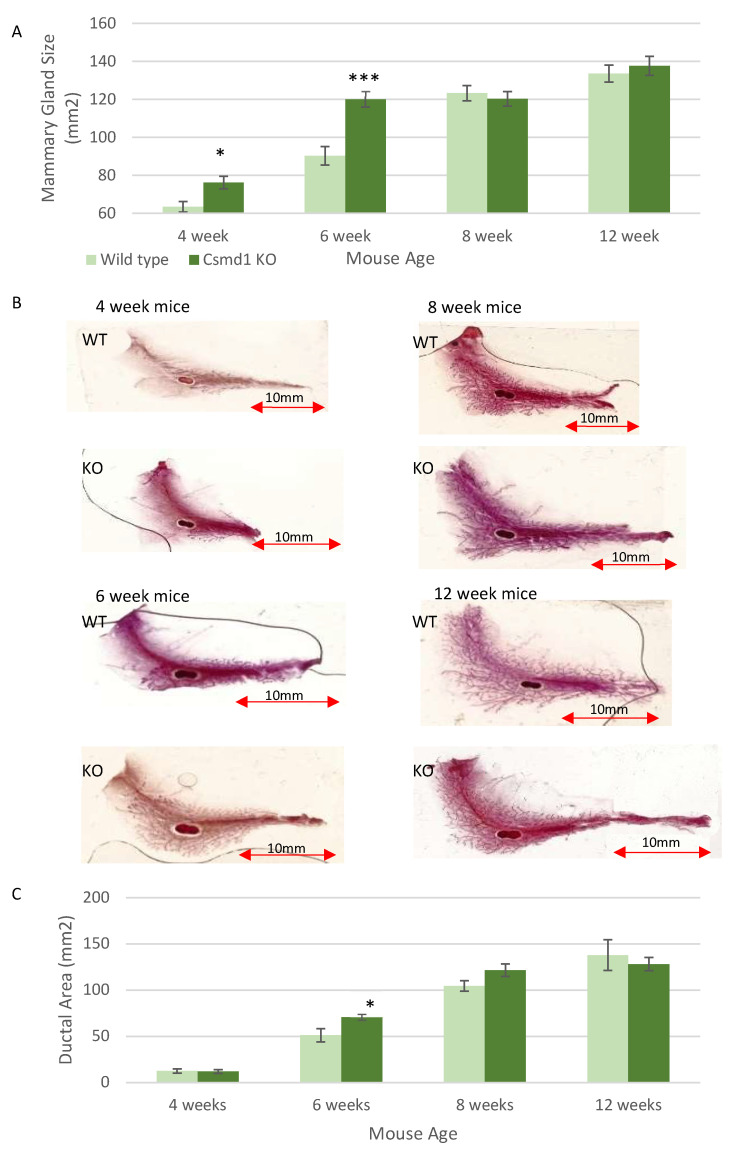
Loss of Csmd1 leads to increased mammary gland size and ductal growth. (**A**) Graph showing the changes in mammary gland size between the WT and Csmd1 KO mice. The area of the abdominal fat pad was measured in each mouse (mm^2^), showing significant changes at 4 and 6 week (* *p* < 0.05 and *** *p* < 0.001). The numbers of animals for each time point included 4-week-old mice (*n* = 12: WT; *n* = 14 KO), 6-week-old mice (*n* = 5: WT; *n* = 11 KO), 8-week-old mice (*n* = 7: WT; *n* = 8 KO), 12-week-old mice (*n* = 7: WT; *n* = 9 KO). (**B**) Whole mount stained mammary glands at all ages (4, 6, 8, 12 weeks) from both WT and KO mice, scale bar 10 mm. The images show the ductal trees within the mammary gland fat pad. (**C**) Graph showing the differences in ductal tree area between the WT and KO mice, (mm^2^), with significant differences noted at 6 weeks (* *p* < 0.05). The numbers of animals for each time point included 4-week-old mice (*n* = 12: WT; *n* = 10 KO), 6-week-old mice (*n* = 5: WT; *n* = 10 KO), 8-week-old mice (*n* = 7: WT; *n* = 7 KO), 12-week-old mice (*n* = 7: WT; *n* = 7 KO).

To explore the changes of *Csmd1* KO on mammary gland development, initially the size of the mammary glands of each female mouse was measured. Male mice were not analysed in this mammary gland study due to the fact that they only develop a rudimentary ductal structure in which any changes caused by *Csmd1* KO may be difficult to be detected. This identified increased mammary gland sizes in both at 4 weeks (*p* < 0.05) and 6 weeks (*p* < 0.001) of age in the KO mice (Figure 2A). However, subsequently there were no significant changes in mammary gland size in either the 8 or 12 week old mice, which had similar sized mammary glands in the KO mice and the WT mice (Figure 2A). Next we explored whether the ductal tree itself shows signs of increased growth. Interestingly, the ductal trees at 4 weeks of age were of a similar size in both the KO and WT mice, despite the increased mammary gland size observed (Figure 2B,C). The ductal tree did show a significant increase in size in the 6 week old KO mice (*p* < 0.05), with the ductal area being slightly larger at 8 weeks in the KO mice, but not significantly so, and then the ductal area was a similar size at 12 weeks in both the KO and WT mice (Figure 2C).

**Figure 3:**
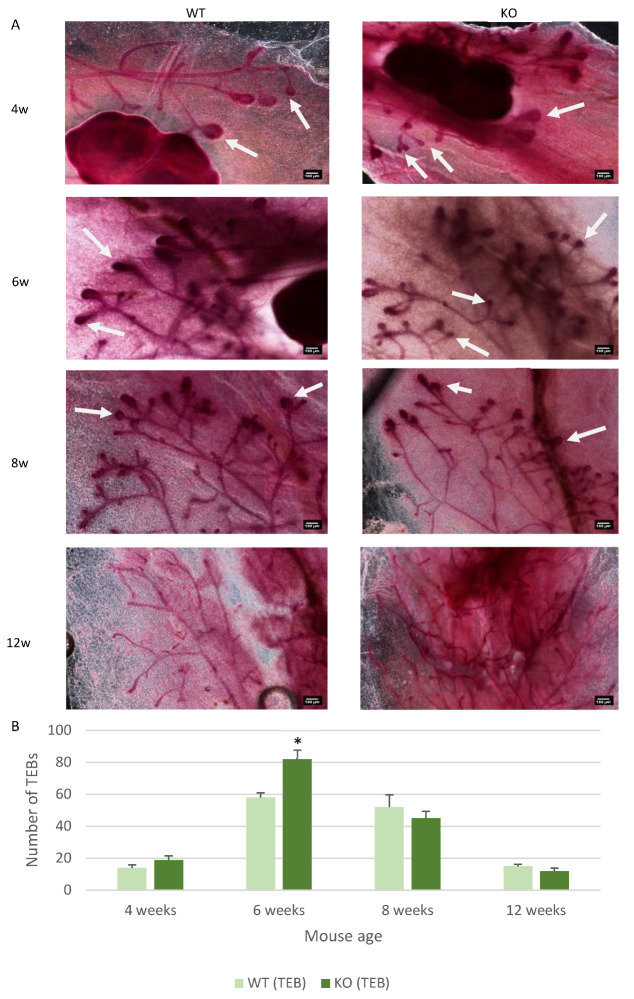
Csmd1 KO leads to increased terminal end bud number during duct development. (**A**) Images of the whole mount stained mammary glands taken at x4 objective. The terminal end buds are identified in the images by the white arrows and the scale is 100 µm. (**B**) Graph depicting the changes in terminal end bud number between the WT and KO mice, changes were observed at 6 weeks of age (* *p* < 0.05). The numbers of animals for each time point included 4-week-old mice (*n* = 12: WT; *n* = 10 KO), 6-week-old mice (*n* = 5: WT; *n* = 10 KO), 8-week-old mice (*n* = 7: WT; *n* = 7 KO), 12-week-old mice (*n* = 7: WT; *n* = 7 KO).

To further understand what could be causing the increased ductal area observed, the number of TEBs was analysed. This is due to the role TEBs play in driving duct invasion and development into the mammary gland fat pad [30]. This identified similar levels of TEB numbers at 4, 8 and 12 weeks of age in the KO and WT mice (Figure 3A,B). However, at 6 weeks of age the number of TEBs identified in each gland was significantly increased (*p* < 0.05) in the KO mice compared to the WT mice (Figure 3B).

This therefore shows that in *Csmd1* KO mice the deficiency of Csmd1 is able to drive increased ductal development at an early stage of puberty (6 weeks). This is demonstrated by increased mammary gland size and ductal area, but also through increased TEB number at this time point.

### 3.2. Csmd1 KO is Able to Generate Changes in Ductal Morphology, along with Altering Mammary Gland Development Factors

**Figure 4:**
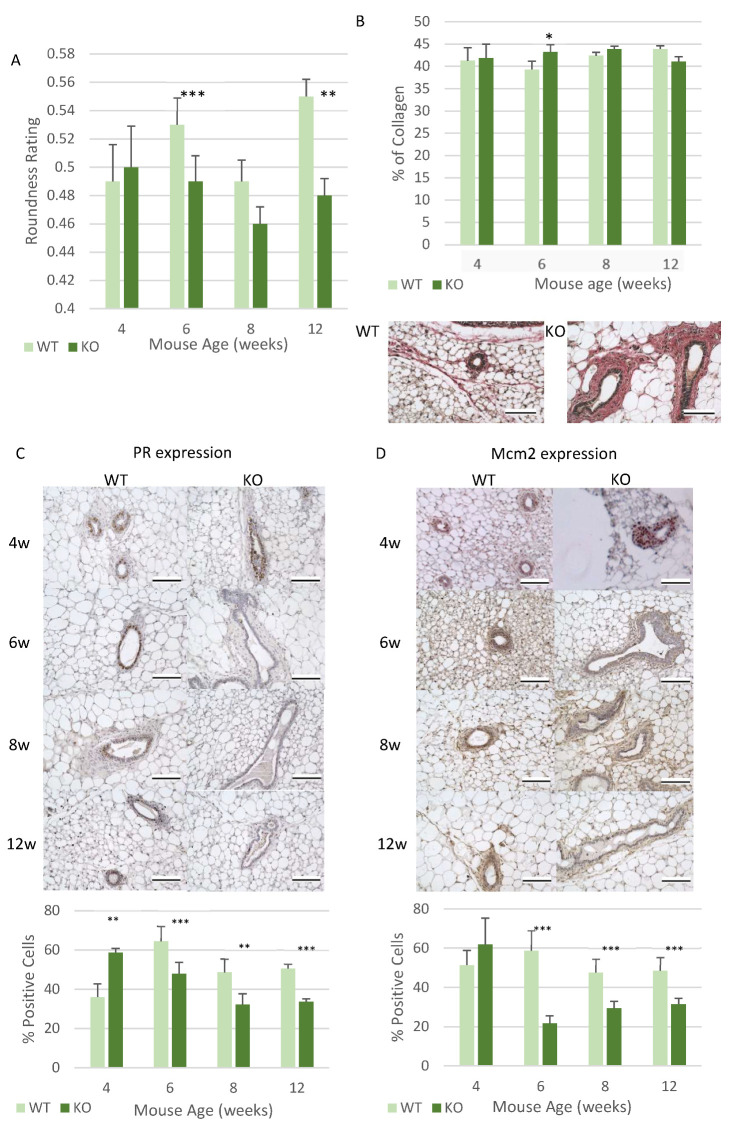
Loss of Csmd1 is able to promote changes in ductal morphology and development factors. (**A**) Graph showing changes in duct roundness, the lumen of the ducts were measured and then analysed using ImageJ software. The roundness rating is between 0 and 1, with 1 being a perfect circle (** *p* < 0.01 and *** *p* < 0.001). The numbers of animals for each time point included 4-week-old mice (*n* = 6 WT; *n* = 6 KO), 6-week-old mice (*n* = 8 WT; *n* = 7 KO), 8-week-old mice (*n* = 7 WT; *n* = 7 KO), 12-week-old mice (*n* = 7 WT; *n* = 7 KO). (**B**) Analysis of the Picro-Sirus Red staining, which is used to stain collagen red. The staining is shown in the images below the graph which depicts ducts at 6 weeks of age in the WT and KO mice, with the red collagen staining located around each duct. The graph shows the percentage of collagen surrounding each duct, which was calculated using ImageJ (* *p* < 0.05) (*n* = 6 WT; *n* = 6 KO), 6-week-old mice (*n* = 8 WT; *n* = 7 KO), 8-week-old mice (*n* = 7 WT; *n* = 7 KO), 12-week-old mice (*n* = 7 WT; *n* = 7 KO) (**C**) The analysis of progesterone receptor (PR) expression, the images show respective examples of the ducts at each time point in both the WT and KO mice. The graph highlights the percentage of cells which positively express PR (** *p* < 0.01 and *** *p* < 0.001). The numbers of animals for each time point included (*n* = 6 WT; *n* = 6 KO), 6-week-old mice (*n* = 8 WT; *n* = 7 KO), 8-week-old mice (*n* = 7 WT; *n* = 7 KO), 12-week-old mice (*n* = 7 WT; *n* = 7 KO). (**D**) Analysis of the level of proliferation occurring in each duct in the WT and KO mice. The images show proliferation in the ducts at all ages in both the WT and KO mice, with the graph showing number of cells actively proliferating (*** *p* < 0.001). The numbers of animals for each time point included 4-week-old mice (*n* = 6 WT; *n* = 6 KO), 6-week-old mice (*n* = 8 WT; *n* = 7 KO), 8-week-old mice (*n* = 7 WT; *n* = 7 KO), 12-week-old mice (*n* = 7 WT; *n* = 7 KO). All images taken using an x40 objective, with all scale bars 100 µm.

Next, any changes at the cellular level during development, along with any changes in regulatory growth patterns, were explored. The morphology of the ducts was analysed to identify changes to the structure of the ducts in the KO mice, to see if they show dysregulated developmental patterns. This was analysed by measuring the roundness of the ducts. Healthy/normal ducts tend to have round/circular ducts with dysregulated ducts producing non-uniform shapes and therefore less rounded ducts. In this study the *Csmd1* KO mice demonstrated a decrease in roundness of ducts at 6 weeks (*p* < 0.001) and 12 weeks (*p* < 0.01), with a slight decrease in roundness observed at 8 weeks (Figure 4A). This therefore indicates that the ducts are undergoing altered ductal morphological changes in the KO mice, leading to irregular development of the ducts. Here the KO mice have a higher number of ducts with irregular shapes, which leads to the decrease in duct roundness observed, whereas in the WT mice there are very few irregular ducts.

Potential changes in factors which effect mammary gland development were also analysed, which included collagen levels, progesterone receptor (PR) expression and proliferation. Collagen was analysed due its role in regulating duct development, and also that it is the most commonly expressed protein within the ECM [34]. The amount of collagen surrounding each duct remained at similar levels at 4, 8 and 12 weeks of age between the KO and WT mice (Figure 4B). However, there was a significantly higher level of collagen deposition around each duct at 6 weeks of age (*p* < 0.05) (Figure 4B). The expression of PR was also analysed since it plays an important role in promoting ductal invasion into the mammary fat pad [35,36]. The KO mice demonstrated significant changes in PR expression at all of the analysed time points. At 4 weeks the KO mice had significantly higher levels of PR expression (*p* < 0.01) compared to the WT mice (Figure 4C). Interestingly, from 6 weeks onwards, the KO mice expressed significantly lower levels of PR than the WT mice (*p* < 0.001 (6/12 weeks) and *p* < 0.01 (8 weeks)) (Figure 4C). When exploring if there were any changes in the number of proliferating cells, it was noticed that a similar expression to that of PR was observed. This meant that there were a higher number of proliferating cells at 4 weeks of age, although in this instance the increase observed did not reach statistical significance (Figure 4D). Then from 6 weeks onwards there was a significant decrease in the number of proliferating cells at each time point (*p* < 0.001) (Figure 4D).

These changes further consolidate that there is a significant change occurring during the early stages of mammary gland development (4–6 weeks).

### 3.3. Csmd1 KO Leads to the Altered Expression Patterns of Mammary Gland Development Associated Proteins

To explore potential mechanisms of action in which Csmd1 could be involved in regulation, the expression patterns of various proteins connected with mammary gland development were analysed.

**Figure 5:**
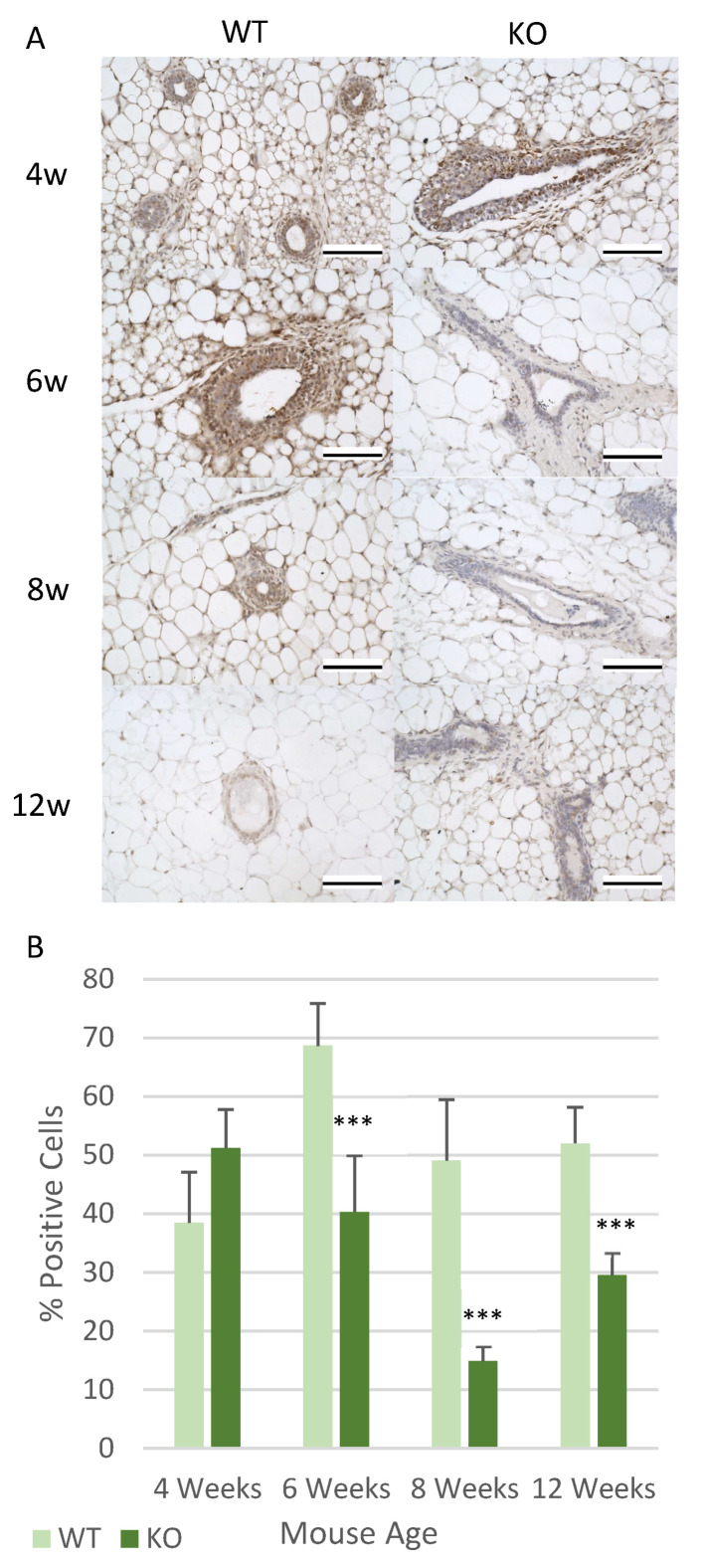
Changes in Stat1 expression are generated by *Csmd1* KO. (**A**) Images showing the expression of Stat1 within the mammary glands ducts at all time points in both the WT and KO mice, stained using IHC (**B**) Graph highlighting the average percentage of cells positively expressing Stat1 between the WT and KO ducts by counting the percentage of positive cells per duct (*** *p* < 0.001). The numbers of animals for each time point included (*n* = 6 WT; *n* = 6 KO), 6-week-old mice (*n* = 8 WT; *n* = 7 KO), 8-week-old mice (*n* = 7 WT; *n* = 7 KO), 12-week-old mice (*n* = 7 WT; *n* = 7 KO). All images were taken using an x40 objective, with all scale bars 100 µm.

One of the proteins analysed was the signal transducer and activator of transcription 1 protein (Stat1), which has been shown to have high levels of expression during early and peak puberty, with these levels then decreasing as the mice reach adulthood [37,38]. In this study higher levels of Stat1 expression were identified at 4 weeks in the KO mice, although this was not a significant increase (Figure 5A). However, from 6 weeks onwards at all time points, statistically significantly lower levels of Stat1 expression were observed in the KO mice compared to the WT mice (*p* < 0.001) (Figure 5B). Interestingly, it was also observed that in the WT mice the peak expression for Stat1 was observed at 6 weeks of age, but in the KO mice the highest level of Stat1 expression was noticed at 4 weeks (Figure 5B). There was also a similar pattern observed in the PR expression analysis (Figure 4C).

**Figure 6:**
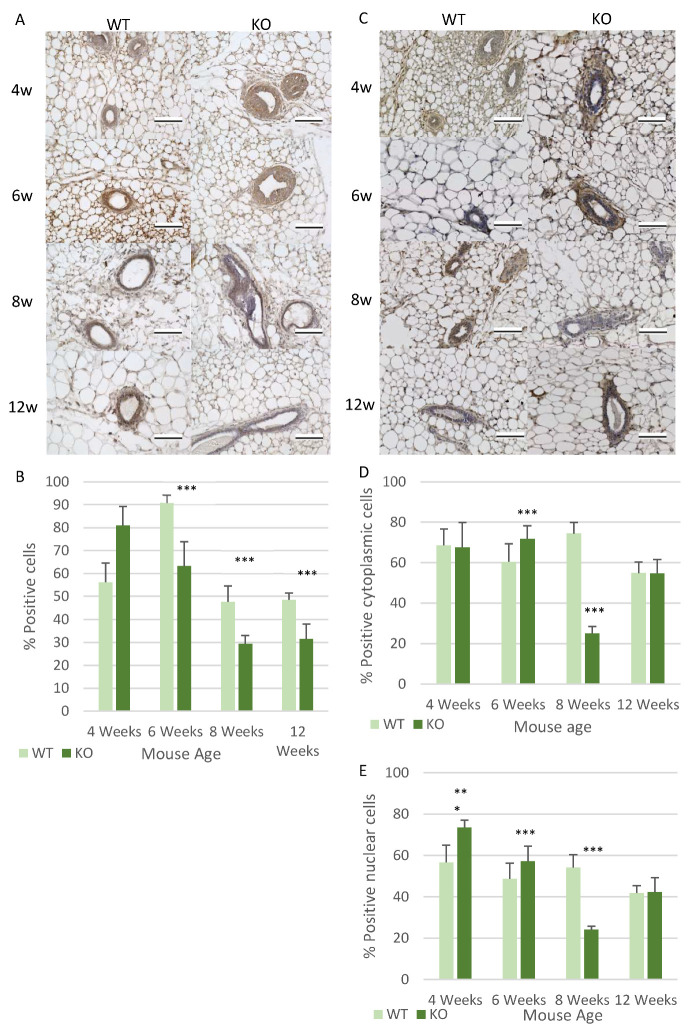
*Csmd1* KO causes changes in the expression of cell adhesion associated proteins. (**A**) Images of Fak expression within the ducts of the mammary glands at all ages in both the WT and KO mice. (**B**) Graph highlighting the changes in Fak expression in each duct at the various ages between the WT and KO mice, with the average expression of Fak per duct being shown (*** *p* < 0.001). The numbers of animals for each time point (*n* = 6 WT; *n* = 6 KO), 6-week-old mice (*n* = 8 WT; *n* = 7 KO), 8-week-old mice (*n* = 7 WT; *n* = 7 KO), 12-week-old mice (*n* = 7 WT; *n* = 7 KO) (**C**) Images of Slug and Snail expression within the ducts of the mammary glands at all ages in both the WT and KO mice. (**D**) Graph highlighting the changes in cytoplasmic levels of Slug and Snail expression at the various ages between the WT and KO mice, with the average expression of Slug and Snail per duct being shown (*** *p* < 0.001). (**E**) Graph highlighting the changes in nuclear Slug and Snail expression at the various ages between the WT and KO mice, with the average expression of Fak per duct being shown (*** *p* < 0.001). The numbers of animals for each time point included (*n* = 6 WT; *n* = 6 KO), 6-week-old mice (*n* = 8 WT; *n* = 7 KO), 8-week-old mice (*n* = 7 WT; *n* = 7 KO), 12-week-old mice (*n* = 7 WT; *n* = 7 KO). All images were taken using an x40 objective, with all scale bars 100 µm.

The changes observed in the ducts ability to infiltrate the mammary fat pad suggests an increase in the invasive properties of the cells in the *Csmd1* KO mice. An important mechanism regulating cell invasion is cell adhesion mediated signalling, consequently the expression of cell adhesion regulating molecules were analysed. These were the focal adhesion kinase (Fak) [39,40] and Slug/Snail signalling [41]. The expression pattern of Fak seemed to show a very similar pattern of expression to that of Stat1, in that at 4 weeks there is a non-significant increase in Fak expression in the KO mice (Figure 6A,B). Followed by a statistically significant decrease in expression at all time points from 6 weeks onwards (*p* < 0.001) (Figure 6B). The highest peaks in expression were observed at 4 weeks in the KO mice and 6 weeks in the WT mice. Both the levels of cytoplasmic and nuclear Slug/Snail expression were measured, revealing a statistically significant higher level of nuclear expression in the KO mice at both 4 and 6 weeks of age (*p* < 0.001), which then significantly decrease at 8 weeks (*p* < 0.001) before the Slug/Snail expression levels become similar to the WT in the adult mice (Figure 6C–E). A slightly different pattern of Slug/Snail cytoplasmic expression was observed with similar levels of expression at 4 weeks of age before Slug/Snail expression is significantly increased at 6 weeks of age in the KO mice (*p* < 0.001) (Figure 6C,D). This was followed by significantly lower Slug/Snail cytoplasmic expression in the KO at 8 weeks (*p* < 0.001), before having similar levels of expression at 12 weeks in the KO and WT mice (Figure 6D). Slug/Snail displays a different expression pattern compared to most of the other proteins analysed, as it seems to have higher Slug/Snail expression at 4 weeks (in the nucleus) in the KO mice which is the same as other proteins analysed, but it also has higher levels of expression at 6 weeks in the KO (Figure 6E). This 6-week change shows an alternative expression pattern for the KO mice. Furthermore the WT expression pattern is also different for Slug/Snail expression as there is no clear observable peak of expression at 6 weeks, instead the expression levels of Slug/Snail remain similar between 4–8 weeks before decreasing at 12 weeks (Figure 6D,E).

**Figure 7:**
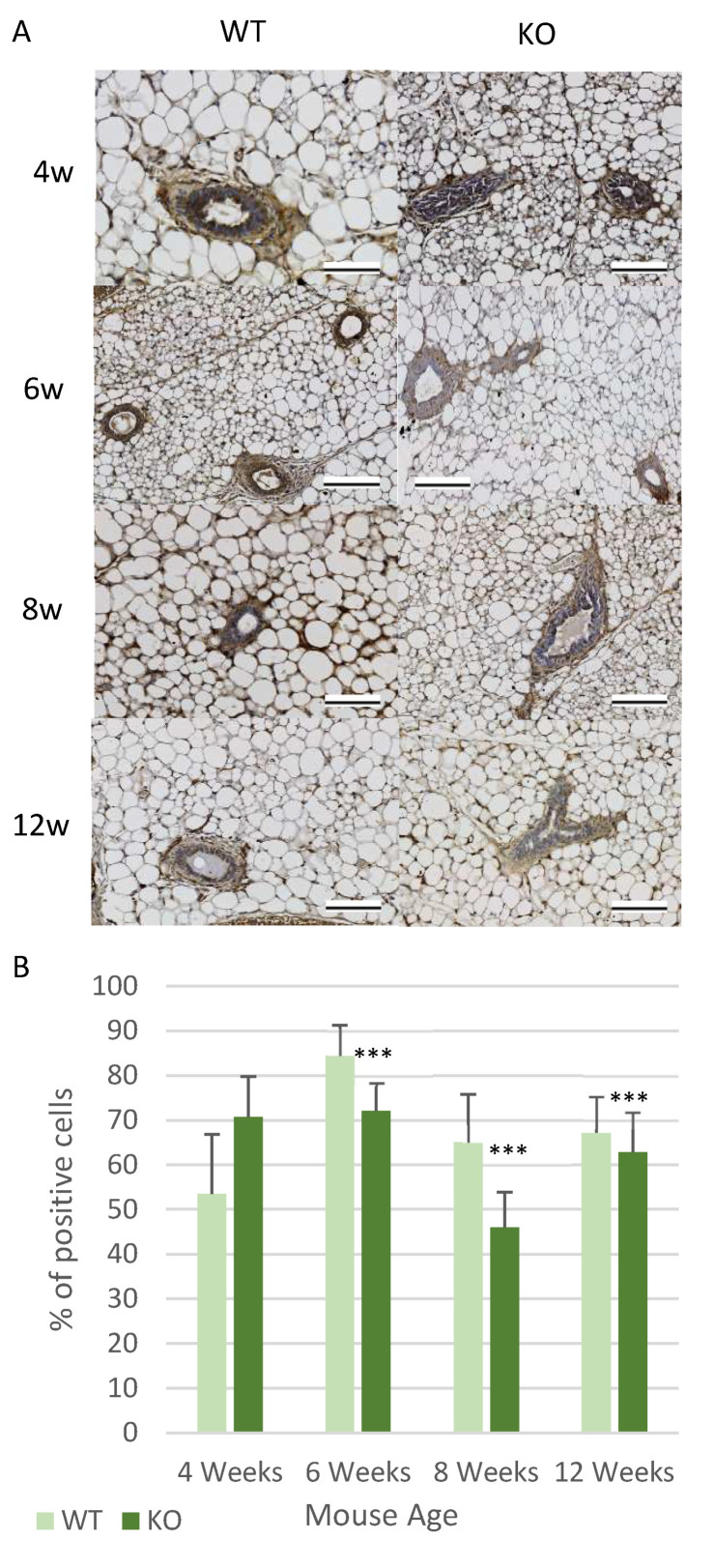
Loss of Csmd1 causing changes in the expression patterns of Pi3k/Akt signaling. (**A**,**B**) Akt expression analysis. (**A**) Images showing the amount of positive Akt expression in the mammary gland ducts at all ages in the WT and KO mice, using IHC staining. (**B**) The graph shows the average percentage of cells which are positively expressing Akt in each duct (*** *p* < 0.001). The numbers of animals for each time point included (*n* = 6 WT; *n* = 6 KO), 6-week-old mice (*n* = 8 WT; *n* = 7 KO), 8-week-old mice (*n* = 7 WT; *n* = 7 KO), 12-week-old mice (*n* = 7 WT; *n* = 7 KO). All images were taken using an x40 objective, with all scale bars 100 µm.

Finally, the expression of the Pi3k/Akt signalling cascade was analysed by measuring the levels of Akt. This signalling pathway was chosen because it is commonly upregulated in cells and cancers, which are undergoing EMT [42,43,44]. In the case of the Akt expression, it showed higher levels of expression at 4 weeks in the KO mice, although this was not a significant increase. However, there was statistically significantly less Akt expression in the KO from 6 weeks onwards (*p* < 0.001) (Figure 7A,B). In contrast to the other proteins analysed there was not a decrease in Akt expression between 4 and 6 weeks of age, as the expression levels remained similar. Instead, the Akt in the KO mice appeared to decrease from 8 weeks (Figure 7B). However, in the WT mice the Akt expression pattern was similar to the patterns previously observed in other proteins, in that it has a noticeable high peak of expression at 6 weeks of age.

**Figure 8:**
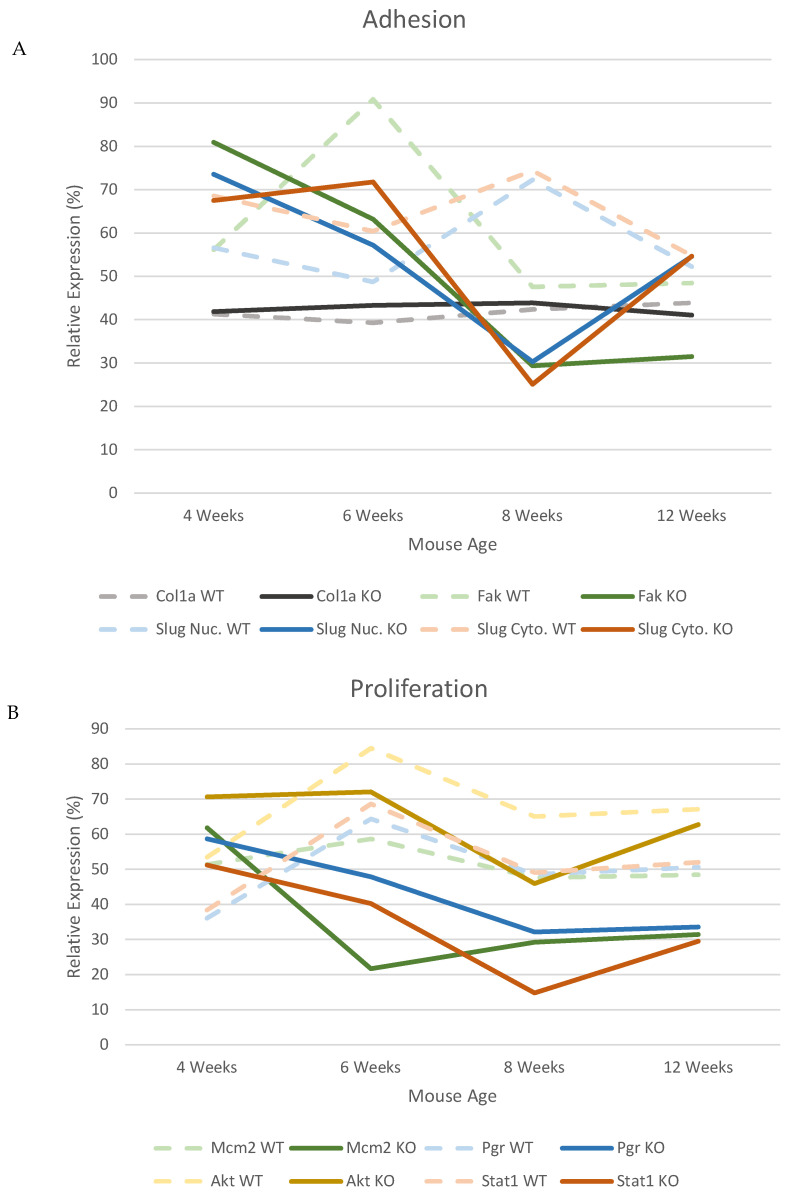
The expression patterns of the proteins analysed show alternative peaks of expression in the *Csmd1* KO mice compared to the WT. The expression levels across all time points (4–12 weeks) are shown to demonstrate how the specific expression of the proteins changes over puberty and also how the patterns vary between the WT and KO mice. (**A**) Graph showing the proteins associated with regulating cell adhesion. (**B**) Graph showing the proteins associated with regulating cell proliferation. In both graphs clear alternative peaks can be observed between the WT, peak of expression commonly at 6 weeks, and the KO mice, peak of expression commonly at 4 weeks.

In the WT mice there is a clear pattern of expression for the majority of proteins, which have their highest level of expression at 6 weeks of age (Figure 8A,B). This therefore highlights that in the WT mice, the time at which the mice are undergoing the largest developmental changes is at 6 weeks, suggesting that this is the peak of puberty in these mice. However in the KO mice this peak in protein expression appears to occur earlier than 6 weeks, with the highest expression levels in the KO mice being at 4 weeks of age. In addition after 6 weeks (Fak, Mcm2, PR, Stat1 and Slug/Snail) or 8 weeks (Akt) in the KO mice there appears to be a large decease in protein levels in the majority of proteins analysed compared to the WT expression levels.

## 4. Discussion

The effects of *Csmd1* KO have been well characterised *in vitro*, with a role for Csmd1 in duct development being identified [13,14,15,22,23] but there have been very few studies exploring the role of Csmd1 in development in vivo [11,14]. Therefore there is a need to expand and confirm the observations seen in vitro in an in vivo environment. In this study the role of Csmd1 in mammary gland development has been explored for the first time in a *Csmd1* KO mouse model.

Throughout this study the *Csmd1* KO mice showed a number of changes during the early stages of female mammary gland development (4–6 weeks). This was initially demonstrated when examining the gross structural changes in the development of the ductal tree, which showed increased mammary gland size and ductal tree area at 6 weeks of age in the KO mice. In addition, this increase in duct development was supported by the observed increase in TEBs at 6 weeks in the KO mice. This therefore suggests that the *Csmd1* KO is able promote a more invasive and expansive ductal tree, with the process being driven by the increased TEB number. This increase in TEB number therefore highlights a potential role for Csmd1 in the regulation of cell proliferation and invasion mechanisms, which have previously been identified [22,23]. This connection is strengthened with changes in proliferation, ECM deposition and in Akt signalling observed in the KO mice [42,45]. Most notably, the higher levels of proliferation and Akt signalling at 4 weeks, followed by the decrease at 6 weeks in the KO mice, which suggests that the earlier high levels of proliferation observed could be able to generate the larger numbers of TEBs observed at 6 weeks in the KO. Potentially, the higher levels of proliferation could be driven by an increase in Akt mediated signalling. Hence the regulation of Akt mediated signalling by Csmd1 could explain the increased action and number of TEBs present. This will then promote a more invasive phenotype allowing for increased fat pad infiltration [46,47].

There were also observed changes in the structure of the ducts in the KO mice, which appeared to lead to dysregulated ductal structure. This dysregulated structure could be caused by a number of factors. Firstly if the level of proliferation is increased from an early age, which was shown in the 4 week KO mice, it could cause the ducts to development more rapidly [46], therefore preventing normal ductal formation and also generating higher numbers of cells, leading to abnormal duct shapes. Furthermore, the increase in proliferation might be a driving mechanism behind the increased TEB number. Secondly, another mechanism behind the changes in duct morphology could be related to alterations in cell polarity [48]. Changes in cell polarity are able to effect lumenogenesis and therefore if this is dysregulated in the KO mice, then the lumens of the ducts will be unable to form, therefore producing the dysregulated duct formation [48]. Any potential changes in cell polarity can be regulated by tight junction function [49], therefore conferring a connection to cell adhesion and invasion mechanisms.

Other factors which are able to promote ductal development are changes in hormone regulation and ECM deposition [34,36]. In this study significant changes were observed between the KO and WT mice in both these factors during the early stages of puberty. The levels of PR are shown to be significantly increased at 4 weeks in the KO mice but then were significantly lower from this point onwards. The ECM appears to only show a significant increase in collagen deposition at 6 weeks of age in the KO mice. The increased level of PR expression per duct suggests increased susceptibility to progesterone in the *Csmd1* KO mice from an early age. This increased level of progesterone mediated action could then be a factor behind increased TEB numbers observed at 6 weeks. This is due to the fact that PR and progesterone action plays a prominent role in promoting TEBs [30,36,50,51]. Interestingly it has been shown that in normal (WT) mice PR expression appears to peak between 6–7 weeks of age, however in our *Csmd1* KO mice this peak appears to occurring noticeably earlier at 4 weeks [36]. This further highlights the potential of *Csmd1* KO mice undergoing more rapid duct development. The increase in ECM deposition observed at 6 weeks is likely to be a by-product of the increased duct development. However it could also be involved in promoting the increased duct development in the KO mice. This is because when higher levels of ECM deposits are noticed it is usually is an identifying factor of a stiffer ECM matrix. This stiffer matrix is able to promote cell-ECM interactions which in turn promotes cell invasion and duct elongation, consequently the increased ECM deposition could also promote the increased ductal growth observed [34,52]. This study demonstrates that *Csmd1* KO causes changes in both hormone regulation and ECM deposition, with these changes leading to a more invasive ductal phenotype [34,53]. The link between reduced CSMD1 expression and a more invasive ductal phenotype is also supported by human breast cancer studies, in which reduced CSMD1 expression was associated with reduced differentiation and increased tumour grade [17].

Due to changes observed in duct invasion and ECM levels we wanted to identify a specific mechanism of action that could be being promoted/causing these changes. One such mechanism is cell adhesion signalling which has been shown to be involved in interacting with the microenvironment to regulate cell invasion [54]. For this reason the cell adhesion regulator proteins Fak [55] and Slug/Snail [56] were analysed to see if this is a potential mechanism of action for Csmd1. Both of these proteins showed increased expression levels at 4 weeks in the KO mice, with the Fak expression becoming lower than the WT levels from 6 weeks. However the levels of Slug/Snail remained higher at 6 weeks in the KO and then reduced to levels lower than the WT from 8 weeks. This suggests that *Csmd1* KO could cause changes in cell adhesion regulation. The potential impact of these changes is that the duct cells in the KO mice are able to generate an increased number of cell-ECM interactions, via increased focal adhesion action [57,58,59]. Since the changes in Fak and Akt expression are similar, this suggests that increased Fak mediated signalling of Akt pathways is occurring in the *Csmd1* KO mice [60,61,62]. In which this signalling cascade then promotes increased cell migration and invasion in the KO mice, via the increased action of focal adhesions [60,61,62].

The expression patterns of Stat1 was determined in this study to corroborate the earlier mammary gland development observed in the *Csmd1* KO mice. Stat1 is an excellent marker for mammary gland development stage because Stat1 demonstrates its highest levels of expression at the beginning of puberty when development of the mammary gland is at its highest, with the levels decreasing into adulthood [37,38]. Consequently by analysing the levels of Stat1 expression it’s possible to determine when the mammary gland is undergoing peak puberty. In this study in the WT mice the peak Stat1 expression is at 6 weeks of age, but in the *Csmd1* KO mice this peak is at 4 weeks of age. This provides rather compelling evidence that the mammary glands in the KO mice are likely to be undergoing more rapid mammary gland development. This concept is supported by changes in expression patterns of the other markers analysed in the study (Mcm2, PR, Fak), they all demonstrate the peak of expression in the WT mice at 6 weeks of age. However in the KO mice the majority of the markers have the highest expression at 4 weeks of age. This would suggest that as the KO mice have higher expression levels of protein expression from an earlier age (4 weeks) they are able to drive the phenotypical changes observed at 6 weeks. Consequently a fully formed ductal tree is observed at 8 weeks of age in the KO mice as there is no change in ductal tree size between 8–12 weeks, whereas in the WT mice this fully formed ductal tree appears at 12 weeks.

## 5. Conclusions

In summary, this study explores for the first time the role of Csmd1 in the process of mammary gland development, leading on from previous in-vitro ductal models [22]. Increased ductal development from an early age was identified in the *Csmd1* KO mice and that this development occurs more rapidly leading to the earlier generation of an adult duct. The changes identified throughout this study identify a strong connection between Csmd1 and the regulation of cell invasiveness, which is potentially controlled via changes in cell adhesion mechanisms. Since Csmd1 is a known tumour suppressor gene it would be interesting to explore the role of Csmd1 in cancer in vivo, to determine if the in-vitro changes observed in this study are also involved in regulating cancer development.

## Figures and Tables

**Table 1:** Antibodies used in this study. Table shows the host species, target area of the protein which the antibody binds and the IHC conditions including stock dilution and the antigen retrieval times (performed in pH6 citrate buffer unless stated).

Antibody (Reference Number)	Host Species	Binding Area of Protein of Interest	IHC Working Conditions (Antigen Retrieval)
Mcm2 (ab4461)	Rabbit	Aa 1–50	1:100 (2 min)
PR (RM-9102-s0)	Rabbit	Aa 412–526	1:400 (2 min)
Stat1 (ab47425)	Rabbit	Aa 694–743	1:300 (3 min)
Fak (ab40794)	Rabbit	Aa 700–800	1:100 (4 min)
Slug/Snail (ab180714)	Rabbit	Aa 236–264	1:200 (2 min)
Akt (ab179463)	Rabbit	Aa 250 to C terminus	1:200 (2 min, pH 9 citrate buffer)
